# Anaemia in the first week may be associated with long-term mortality among critically ill patients: propensity score-based analyses

**DOI:** 10.1186/s12873-023-00806-w

**Published:** 2023-03-22

**Authors:** I-Hung Lin, Pei-Ya Liao, Li-Ting Wong, Ming-Cheng Chan, Chieh-Liang Wu, Wen-Cheng Chao

**Affiliations:** 1 Division of Chest Medicine, Department of Internal Medicine, Taichung Veterans General Hospital, Taichung, Taiwan; 2 Department of Medical Research, Taichung Veterans General Hospital, Taichung, Taiwan; 3 Division of Critical Care and Respiratory Therapy, Department of Internal Medicine, Taichung Veterans General Hospital, Taichung, Taiwan; 4 College of Science, Tunghai University, Taichung, Taiwan; 5 Department of Critical Care Medicine, Taichung Veterans General Hospital, Taichung, Taiwan; 6 Department of Post-Baccalaureate Medicine, College of Medicine, National Chung Hsing University, Taichung, Taiwan; 7 Department of Industrial Engineering and Enterprise Information, Tunghai University, Taichung, Taiwan; 8 Artificial Intelligence Studio, Taichung Veterans General Hospital, Taichung, Taiwan; 9 Department of Automatic Control Engineering, Feng Chia University, Taichung, Taiwan; 10 Big Data Center, Chung Hsing University, Taichung, Taiwan; 11 Taichung Veterans General Hospital, No, 1650, Section 4, Taiwan Boulevard, Xitun District, Taichung City, 40705 Taiwan

**Keywords:** Anaemia, Long-term outcome, Critical illness, Propensity score

## Abstract

**Background:**

Anaemia is highly prevalent in critically ill patients; however, the long-term effect on mortality remains unclear.

**Methods:**

We retrospectively included patients admitted to the medical intensive care units (ICUs) during 2015–2020 at the Taichung Veterans General Hospital. The primary outcome of interest was one-year mortality, and hazard ratios (HRs) with 95% confidence intervals (CIs) were determined to assess the association. We used propensity score matching (PSM) and propensity score matching methods, including inverse probability of treatment weighting (IPTW) as well as covariate balancing propensity score (CBPS), in the present study.

**Results:**

A total of 7,089 patients were eligible for analyses, and 45.0% (3,189/7,089) of them had anaemia, defined by mean levels of haemoglobin being less than 10 g/dL. The standardised difference of covariates in this study were lower than 0.20 after matching and weighting. The application of CBPS further reduced the imbalance among covariates. We demonstrated a similar association, and adjusted HRs in original, PSM, IPTW and CBPS populations were 1.345 (95% CI 1.227–1.474), 1.265 (95% CI 1.145–1.397), 1.276 (95% CI 1.142–1.427) and 1.260 (95% CI 1.125–1.411), respectively.

**Conclusions:**

We used propensity score-based analyses to identify that anaemia within the first week was associated with increased one-year mortality in critically ill patients.

**Supplementary Information:**

The online version contains supplementary material available at 10.1186/s12873-023-00806-w.

## Background

Anaemia is a highly prevalent issue with deleterious impacts among critically ill patients [1, 2]. It is estimated that approximately 70% of patients who were admitted to the intensive care unit (ICU) experienced anaemia, which may result from phlebotomy, occult gastrointestinal bleeding, erythropoietin deficiencies, low iron levels, and coagulopathy [1, 3, 4]. Accumulating evidence have shown that anaemia in critically ill patients was associated with deleterious short-term adverse outcomes, including prolonged hospital length of stay, ventilator day, and increased hospital mortality [2, 5, 6]. Notably, anaemia appears to be a lasting issue given the current critical care strategy with restrictive red blood cell transfusion [4, 7, 8]. Recent longitudinal studies have further shown that anaemia may be persistent for nearly one year after critical illness [9, 10]. A number of studies have implicated anaemia with persistent inflammation and catabolism in patients who survived from critical illness, so-called chronic critical illness or post-intensive care syndrome [11, 12]. There is a research niche to explore the association between anaemia and long-term mortality in critically ill patients. Notably, the association between anaemia and mortality might be difficult to be clarified given the existence of a number of critical care relevant confounders in real-world data [13, 14]. In this study, we used both the critical care database at Taichung Veterans General Hospital (TCVGH) as well as the nationwide death-registry file in Taiwan to investigate the prevalence of anaemia within the first week and to explore the association between anaemia and long-term mortality in critically ill patients using propensity score-based statistical analyses.

## Methods

### Ethical approval

This study was approved by the Institutional Review Board at the Taichung Veterans General Hospital (SE20249B-1), and informed consent was waived, given that all of the data were de-identified data.

### Patient population and definition of anaemia

This retrospective cohort study enrolled consecutive critically ill patients at TCVGH, a referral centre with approximately 1,600 beds in central Taiwan, between 2015 and 2020. Among patients with more than one ICU admission, the first ICU admission was used as the index ICU admission. Subjects without data on haemoglobin level in the first week of ICU admission were excluded from analyses. The presence of anaemia was defined by the mean level of week-1 haemoglobin lower and higher/equal than 10 g/dL given that 7–9 g/dL is currently recommended target level of haemoglobin in critical care [4, 8, 9, 15].

### Primary outcome

The outcome of interest in the present study was the time to all-cause mortality. The date of death was obtained from the death registration profile of the National Health Insurance Database (NHID) in Taiwan [16]. In brief, Taiwan has implemented a compulsory National Health Insurance (NHI) program since 1995 with nearly total coverage of the population in Taiwan; therefore, we think the date of death in this study should be highly accurate.

### Definition of the covariate

We used the critical care database at TCVGH to retrieve critical data relevant variables, consisting of demographic data, laboratory data, comorbidities in accordance with the Charlson Comorbidity Index (CCI) [17], Acute Physiology and Chronic Health Evaluation (APACHE) II score, presence of shock defined by the use of vasopressor, days of receiving mechanical ventilation, and managements including blood transfusion during admission.

### Statistical analyses

The continuous data were presented as means ± standard deviation, and the categorical data were presented as numbers (percentages). Kaplan-Meier curves in patients whose week-1 haemoglobin was higher or lower than 10 g/dL were plotted. A Cox proportional hazards model was constructed after testing the proportional hazard assumption based on the Schoenfeld residuals [18]. We then determined hazard ratios (HRs) and 95% confidence intervals (CIs) for the overall mortality after adjusting potential confounders, including age, sex, comorbidities, and the other potential cofounders. Statistical analyses were conducted by R (3.6.0), and the level of significance was 0.05.

### Propensity score-based analyses

We used propensity score matching (PSM) and weighting methods, consisting of inverse probability of treatment weight (IPTW) as well as covariate balancing propensity score (CBPS) to explore the association between week-1 anaemia and the long-term overall mortality among enrolled patients [19, 20]. The optimal nearest neighbour matching algorithm was used in PSM, and the calliper distance of the standard mean difference was set at 0.20. In brief, PSM was designed for nonexperimental causal studies by reducing the multidimensional covariate space for the probability of week-1 anaemia or not in the present study into one dimension. However, a number of cases might be excluded from analyses due to the lack of matched control subjects, and the restricted sub-population might raise the concern that the selected population might be distinct from the intended population [19]. Therefore, propensity score weighting methods, including IPTW, have been proposed to enrol the whole population; however, the high weight at the tails of the propensity score distribution could reduce the balance among covariates [21, 22]. Recently, CBPS is increasingly applied given that CBPS enrol the whole population through weighting as well as optimising the covariate balance [20, 23].

### Subgroup analysis

We employed the Wald test to measure the significance of the modification effect of variables, including age, sex, renal disease, liver disease, pulmonary disease, the severity of critical illness, blood transfusion, and haemoglobin-associated laboratory data.

## Results

### Characteristics of the enrolled critically ill patients and the propensity score-matched population

**Fig. 1:**
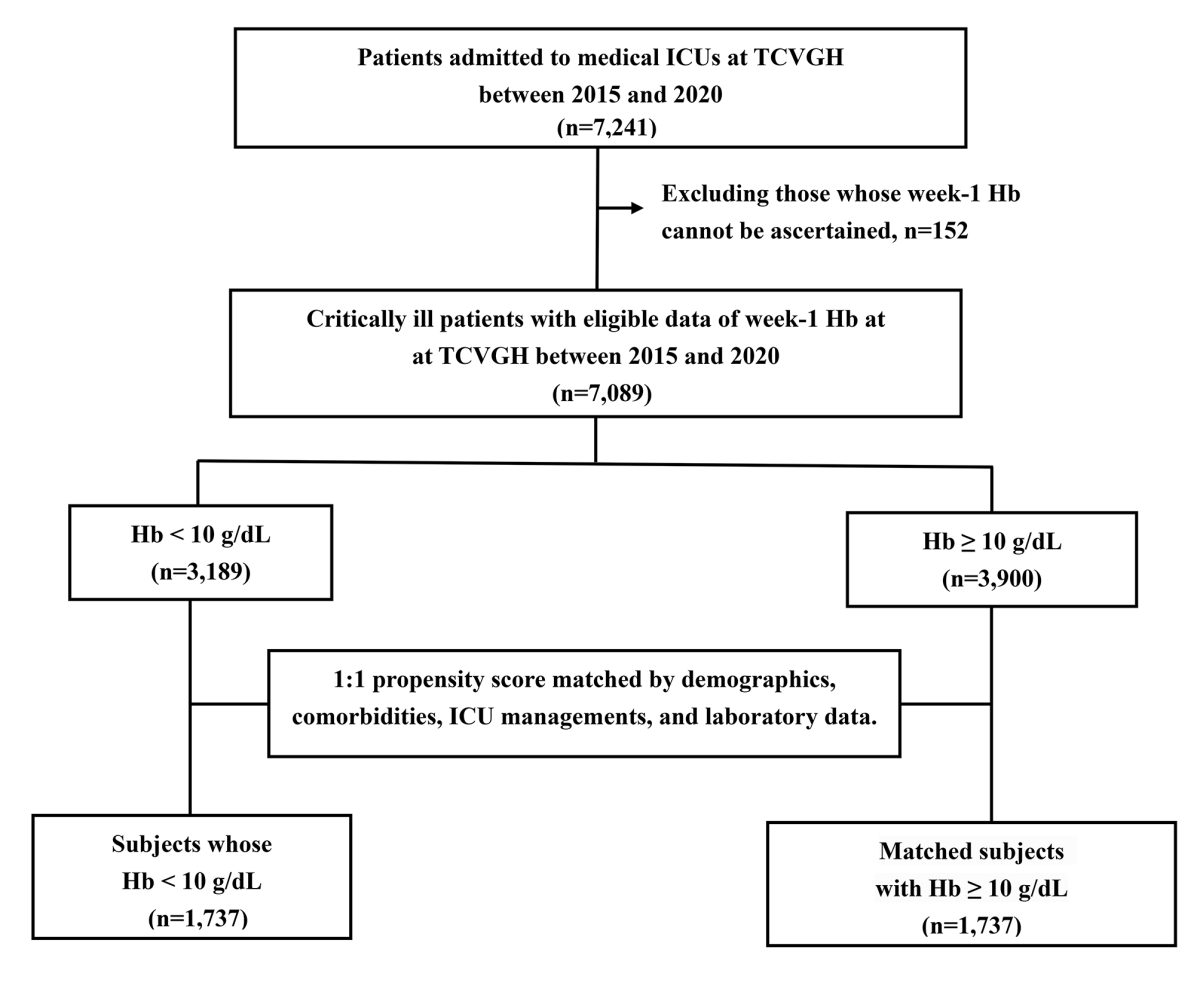
**Flowchart of subject enrollment** Abbreviations: ICUs, intensive care units; TCVGH, Taichung Veterans General Hospital; Hb, Haemoglobin (g/dL)

Figure 1 illustrates the subject enrollment process of the original population (n = 7,089) and propensity core-matched population (n = 3,474) (Fig. 1). A total of 7,089 patients were eligible for analyses, and 45.0% (3,189/7,089) of them had anaemia. Patients with anaemia were older (67.5 ± 16.1 vs. 64.6 ± 15.9 years), had lower body mass index (BMI) (23.8 ± 4.6 vs. 24.6 ± 4.7), were less likely to be male (58.9% vs. 68.1%), had more comorbidities compared those without anaemia. The critical illness-associated variables, including APACHE II score, presence of shock, presence of sepsis, use of mechanical ventilation, leukocytosis, thrombocytopenia, low level of albumin and elevated level of blood urea nitrogen, were more severe in patients with anaemia than those without anaemia. After the PSM, most of variables were comparable between the two groups, although the anaemia group remained has a slightly increased proportion of renal diseases, haematological malignancy, and liver diseases (Table 1).

**Table 1:** Characteristics between the patients categorised by week-1 level of haemoglobin in the primary cohort and propensity score-matched cohort

	Before PSM				1:1 PSM		
	Hb ≥ 10	Hb < 10	SMD		Hb ≥ 10	Hb < 10	SMD
	n = 3,900	n = 3,189			n = 1,685	n = 1,685	
**Basic characteristics**							
Age, years	64.6 ± 15.9	67.5 ± 16.1	0.186		68.1 ± 15.6	67.2 ± 16.5	0.058
Sex (male)	2656 (68.1%)	1879 (58.9%)	0.192		1085 (62.5%)	1085 (62.5%)	< 0.001
Body mass index	24.6 ± 4.7	23.4 ± 4.6	0.257		23.4 ± 4.6	23.5 ± 4.6	0.020
**Comorbidities**							
Diabetes mellitus	1200 (30.8%)	1148 (36%)	0.111		603 (34.7%)	613 (35.3%)	0.012
Myocardial infarction	1283 (32.9%)	481 (15.1%)	0.427		313 (18%)	322 (18.5%)	0.013
Congestive heart failure	599 (15.4%)	521 (16.3%)	0.027		309 (17.8%)	284 (16.4%)	0.038
Valvular heart disease	168 (4.3%)	125 (3.9%)	0.020		4 (0.5%)	4 (0.5%)	< 0.001
Dementia	180 (4.6%)	191 (6%)	0.061		122 (7%)	109 (6.3%)	0.030
Cerebrovascular disease	923 (23.7%)	563 (17.7%)	0.149		356 (20.5%)	341 (19.6%)	0.022
Hemiplegia	114 (2.9%)	51 (1.6%)	0.089		38 (2.2%)	36 (2.1%)	0.008
Peripheral vascular disease	230 (5.9%)	194 (6.1%)	0.008		118 (6.8%)	118 (6.8%)	< 0.001
Renal disease	562 (14.4%)	1209 (37.9%)	0.555		412 (23.7%)	476 (27.4%)	0.085
COPD	261 (6.7%)	155 (4.9%)	0.079		118 (6.8%)	98 (5.6%)	0.048
Lymphoma/leukaemia	80 (2.1%)	304 (9.5%)	0.324		74 (4.3%)	113 (6.5%)	0.100
Metastatic solid tumour	475 (12.2%)	727 (22.8%)	0.282		358 (20.6%)	369 (21.2%)	0.016
Rheumatic disease	126 (3.2%)	229 (7.2%)	0.179		91 (5.2%)	94 (5.4%)	0.008
Peptic ulcer	303 (7.8%)	466 (14.6%)	0.218		208 (12%)	210 (12.1%)	0.004
Mild liver disease	322 (8.3%)	548 (17.2%)	0.270		228 (13.1%)	264 (15.2%)	0.059
Moderate or severe liver disease	74 (1.9%)	246 (7.7%)	0.274		68 (3.9%)	102 (5.9%)	0.091
**Severity and managements**							
APACHE II score	22.4 ± 5.6	26.3 ± 6.6	0.651		24.4 ± 6	24.8 ± 6	0.067
Presence of sepsis	371 (9.5%)	694 (21.8%)	0.342		345 (19.9%)	345 (19.9%)	< 0.001
Presence of shock	1462 (37.5%)	2055 (64.4%)	0.560		1023 (58.9%)	1023 (58.9%)	< 0.001
Receiving mechanical ventilation	1435 (36.8%)	2042 (64%)	0.566		1041 (59.9%)	1039 (59.8%)	0.002
Receiving RBC transfusion	638 (16.4%)	2432 (76.3%)	1.503		495 (28.5%)	1213 (69.8%)	0.908
RBC transfusion (units)	2.8 ± 1.1	2.4 ± 0.9	0.409		2.9 ± 1.1	2.5 ± 1.1	0.377
**Laboratory data**							
White blood cell count (10^3^/µl)	10.6 ± 5.3	12.6 ± 5.7	0.170		12.0 ± 6.3	12.2 ± 16.2	0.016
Platelet (10^3^/µL)	210.9 ± 87.4	159.3 ± 108.9	0.523		189.2 ± 94.7	180.8 ± 117.4	0.079
Albumin (mg/dL)	3.6 ± 0.8	2.9 ± 0.7	1.063		3.1 ± 0.6	3.1 ± 0.7	0.045
Blood urea nitrogen (mg/dL)	24.5 ± 18.3	48 ± 33.6	0.870		33.1 ± 23.2	38.4 ± 29.8	0.195
Creatinine (mg/dL)	1.4 ± 1.6	2.9 ± 2.8	0.620		1.9 ± 2.2	2.3 ± 2.6	0.139
**One-year mortality**	988 (25.3%)	1867 (58.5%)	0.715		732 (42.1%)	906 (52.2%)	0.202

Data are shown as mean ± standard deviation and number (percentages). Abbreviations: PSM, propensity score-matching; SMD, standard mean difference; Hb, hemoglobulin (g/dL); COPD, chronic obstructive pulmonary disease; RBC, red blood cell.

### Association between anaemia and one-year mortality

**Figure 2:**
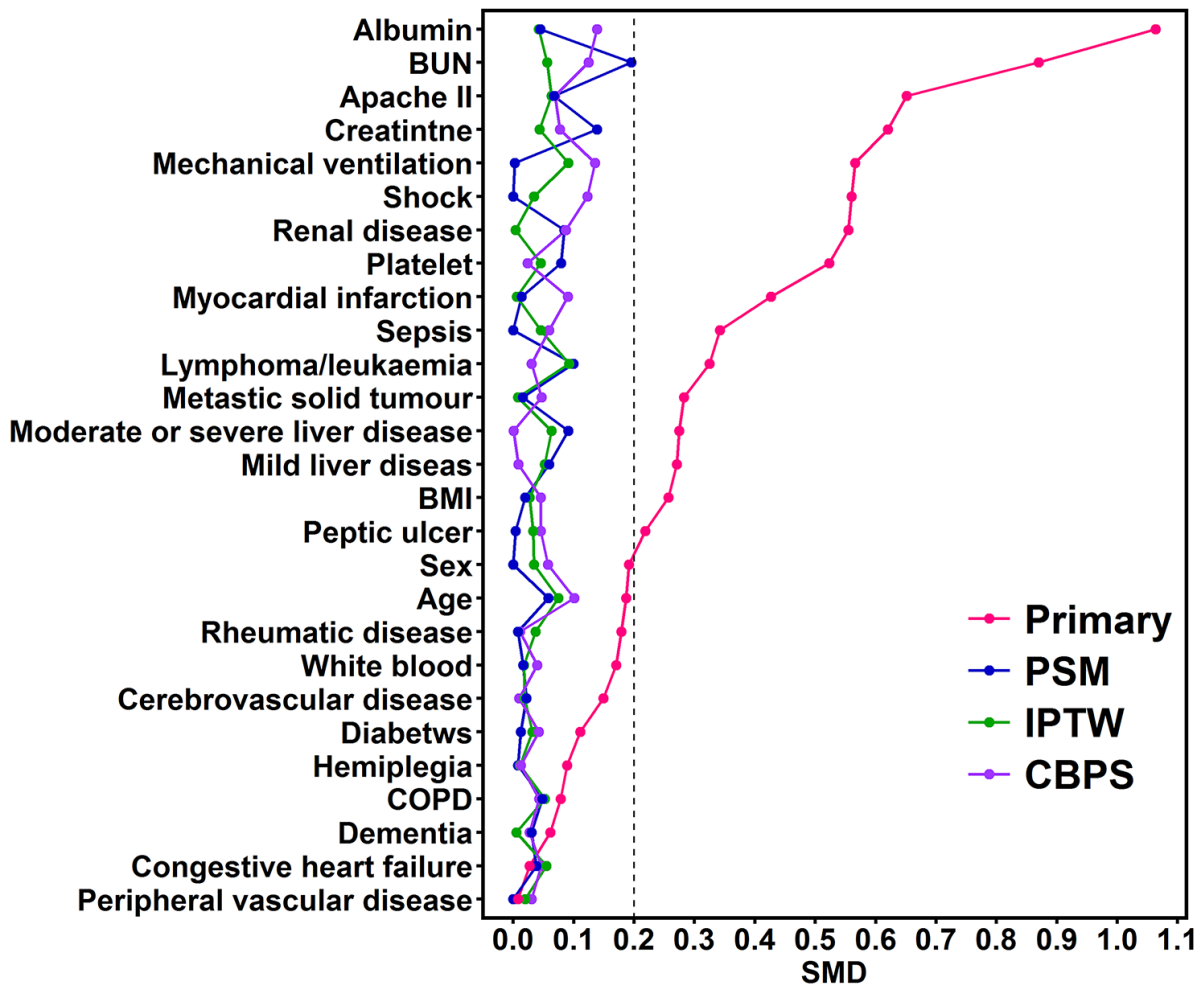
**The standardised mean difference between the patients whose week-1 haemoglobin were higher and lower than 10 g/dL in each cohort.** Variables were ranked by the SMD. Abbreviations: SMD, standard mean difference; PSM, propensity score matching; IPTW, inverse probability of treatment weighting; CBPS, covariate balancing propensity score; APACHE, acute physiology and chronic health evaluation; BUN, blood urea nitrogen; BMI, body mass index; COPD, chronic obstructive pulmonary disease

Figure 2 illustrates the matching quality that was demonstrated by the measurement of the standardised mean difference (SMD) of variables between the two groups in the four distinct cohorts (Fig. 2). In the original population, several variables between patients with and without anaemia were apparently imbalanced. After matching as well as weighting, the aforementioned variables tended to be balanced in both PSM and IPTW, with the SMD lower than 0.20. The application of CBPS further reduced the imbalance of covariates between the two groups. We then focused on investigating the association between anaemia and one-year mortality among enrolled subjects in the aforementioned four populations. We confirmed the fulfilment of the proportional hazard assumption and then adjusted the covariates, including age, sex, and comorbidities in model 2, disease severity and management in model 3, and laboratory findings in model 4 (Supplemental Fig. 1 and Table 2). Table 2 summarises the consistent association between anaemia and risk for one-year mortality in the four populations. The adjusted HRs in the original PSM, IPTW and CBPS populations were 1.345 (95% CI 1.227–1.474), 1.265 (95% CI 1.145–1.397), 1.276 (95% CI 1.142–1.427) and 1.260 (95% CI 1.125–1.411), respectively.

**Table 2:** Cox proportional hazard regressions for estimation of the association between level of week-1 haemoglobin lower than 10 g/dL and one-year mortality in critically ill patients

	Primary population			PSM population			IPTW population			CBPS populaiton		
	HR (95%CI)	p value		HR (95%CI)	p value		HR (95%CI)	p value		HR (95%CI)	p value	
**Model 1**	2.950 (2.730–3.187)	< 0.001		1.337 (1.213–1.474)	< 0.001		1.241 (1.076–1.433)	0.003		1.454 (1.304–1.622)	< 0.001	
**Model 2**	2.175 (1.998–2.368)	< 0.001		1.334 (1.209–1.471)	< 0.001		1.253 (1.103–1.424)	0.001		1.356 (1.216–1.513)	< 0.001	
**Model 3**	1.706 (1.564–1.861)	< 0.001		1.308 (1.185–1.443)	< 0.001		1.271 (1.126–1.436)	< 0.001		1.318 (1.178–1.476)	< 0.001	
**Model 4**	1.345 (1.227–1.474)	< 0.001		1.265 (1.145–1.397)	< 0.001		1.276 (1.142–1.427)	< 0.001		1.260 (1.125–1.411)	< 0.001	

Model 1. Unadjusted

Model 2. Adjusted for demographic data and comorbidities listed in Table 1.

Model 3. Adjusted for variables in model 2 and critical illness severity/management, including APACHE II score, sepsis, shock and use of mechanical ventilation.

Model 4. Adjusted for variables in model 3 and laboratory data, including white blood cell count, platelet count, albumin, blood urea nitrogen, and creatinine.

Abbreviations: PSM, propensity score matching; IPTW, inverse probability of treatment weighting; CBPS, covariate balancing propensity score; HR, hazard ratio; CI, confidence interval.

### Analyses of interactive effects among patients in the PSM cohort

We further conducted subgroup analyses to explore the potential interaction effect of factors, particularly comorbidities that remained imbalanced in the PSM cohort and haemoglobin-associated variables (Table 3). We found that the majority of variables had no modification effect except for moderate/severe liver disease (p = 0.004). The association between anaemia within the first week and one-year mortality mainly existed in patients without liver disease (HR 1.361, 95% CI 1.230–1.506), whereas the association no longer exist in patients with moderate/severe liver disease (HR 0.806, 95% CI 0.560–1.159).

**Table 3:** Stratified analyses for modification effect on the association between level of week-1 haemoglobin and one-year mortality among the 3,440 subjects in the PSM cohort

Variables	Subgroup	Number of patients	Events (death)	HR ratio (95%CI)	p value	p for interaction
**Age** (years)						0.586
	< 65	1388	612	1.367 (1.164–1.605)	< 0.001	
	≥ 65	2086	1026	1.325 (1.172–1.498)	< 0.001	
**Sex**						0.168
	Female	1304	531	1.210 (1.020–1.435)	0.028	
	Male	2170	1107	1.415 (1.256–1.593)	< 0.001	
**Renal disease**						0.588
	No	2586	1268	1.328 (1.189–1.483)	< 0.001	
	Yes	888	370	1.433 (1.163–1.765)	0.001	
**COPD**						0.167
	No	3258	1531	1.361 (1.230–1.506)	< 0.001	
	Yes	216	107	1.031 (0.705–1.508)	0.877	
**Lymphoma/leukaemia**						0.575
	No	3287	1513	1.316 (1.189–1.456)	< 0.001	
	Yes	187	125	1.420 (0.985–2.048)	0.060	
**Moderate or severe liver disease**						0.004
	No	3304	1519	1.361 (1.230–1.506)	< 0.001	
	Yes	170	119	0.806 (0.560–1.159)	0.245	
APACHE II score*						0.765
	≤ 23.00	737	275	1.388 (1.095–1.760)	0.007	
	> 23.00	2737	1363	1.315 (1.182–1.463)	< 0.001	
**Blood transfusion**						0.248
	No	1766	688	1.219 (1.039–1.429)	0.015	
	Yes	1708	950	1.082 (0.939–1.246)	0.275	
**Platelet** (10^3^/µL)*						0.128
	< 173.8	1737	990	1.359 (1.197–1.542)	< 0.001	
	≥ 173.8	1737	648	1.187 (1.017–1.384)	0.029	
**Blood urea nitrogen** (mg/dL)*						0.799
	< 27.0	1707	691	1.326 (1.142–1.540)	< 0.001	
	≥ 27.0	1767	947	1.279 (1.124–1.455)	< 0.001	
**Creatinine** (mg/dL)^*^						0.699
	< 1.2	1660	700	1.289 (1.111–1.494)	0.001	
	≥ 1.2	1814	938	1.305 (1.145–1.486)	< 0.001	

^*^Stratified by median level. COPD, chronic obstructive pulmonary disease; APACHE, acute physiology and chronic health evaluation.

## Discussion

Anaemia is highly prevalent in critically ill patients, but the long-term mortality impact remains unclear. We used propensity score-based matching as well as weighting methods to address the association between anaemia within the first week and long-term mortality in critically ill patients. We identified that week-1 haemoglobin lower than 10 g/dL was associated with an increased risk of one-year mortality, and the association was robust in distinct propensity score-based methods consisting of PSM, IPTW and CBPS. Our findings point out the previously ignored long-term effect of anaemia and indicate that anaemia can be used for long-term mortality risk stratification in critically ill patients.

Anaemia is a prevalent and lasting issue in critically ill patients. Anaemia may result from complex critical illness-associated predisposing factors, including frequent phlebotomy, coagulopathy, blood loss, persistent/dysregulated inflammation, impaired erythropoietic response, kidney injury, and nutritional deficiencies [4, 24, 25]. Moreover, anaemia appears to be a lasting issue among critically ill patients, given that conservative red blood cell transfusion with a restrictive transfusion threshold (7 g/dL) is currently recommended based on the increasing evidence to show potentially detrimental effects of blood transfusion on the short-term outcome in critically ill patients [4, 8, 26]. In the present study, we found that 45.0% (3,189/7,089) of critically ill patients had anaemia, and the relatively high prevalence of anaemia might result from that we used haemoglobin level lower than 10 g/dL to define anaemia. Similarly, Warner et al. recently analysed haemoglobin levels not only during hospitalisation but also 12 months after discharge in 6,901 critically ill patients and found that the prevalence of mild anaemia, defined by haemoglobin level lower than 10 g/dL, at 3-month, 6-month and 12-month after discharge were 56%, 52% and 45%, respectively [9]. Moreover, Warner et al. found that rates of recovery from anaemia at 12 months in patients with mild (10.0–12 g/dL), moderate (8.0–10.0 g/dL) and severe anaemia (< 8.0 g/dL) were 58%, 39% and 24%, respectively [9]. These evidence suggest that anaemia is a lasting issue in critical care and highlight the need to study the long-term effect of anaemia in critically ill patients.

Recently, accumulating studies have used big real-world data to explore critical care-associated issues without large-scale randomised control trials [13, 14]. For example, critical care databases, including Medical Information Mart for Intensive Care (MIMIC) and eICU Collaborative Research Database (eICU-CRD), have been widely used to investigate crucial issues with respect to critical care [27, 28]. However, long-term mortality, a high-priority issue in critical care, cannot be assessed in the aforementioned critical care databases, given that the majority of open data are unidentifiable data that cannot be linked with the other databases [29, 30]. In the present study, the nationwide coverage of compulsory national health insurance in Taiwan enables us to link the critical care database at TCVGH with the Taiwanese NHID and to ascertain the long-term mortality among enrolled subjects. But, the existence of confounders is inevitable in real-world data, and we think it would be imprudent to draw a conclusion without the strenuous effort to mitigate the impact of confounders in real-world data [13, 31]. Collectively, real-world critical care databases are valuable for exploring crucial issues in critically ill patients, but additional efforts are required to mitigate the effect of confounders in real-world data.

In this study, we used three distinct propensity score-based methods to investigate the independent association between anaemia within the first week and one-year mortality in critically ill patients. PSM is a widely used PS-based method to obtain two comparable subgroups through matching; however, a highly stringent matching process in PSM may exclude the high number of enrolled subjects and might restrict the generalizability; therefore, we choose 0.20 as the set calliper distance of the standard mean difference in the present study [19]. Intriguingly, we noted that the strength of association between anaemia within the first week and one-year mortality in the PSM cohort tends to be lower than the strength of association in the primary cohort, IPTW cohort and CBPS (Table 2). We postulated that excluding nearly 50% of the original population during the process of matching might lead to a slight difference in the strength of association despite the similar trend of association (Supplemental Fig. 2). In addition to the matching method, we also employed weighting methods, including IPTW and CBPS, in this study to explore the independent association between anaemia within the first week and one-year mortality among the whole enrolled subjects with a critical illness [21, 22]. In IPTW, all of the subjects were included in analyses, and the PS was used as weights in the analyses to minimise the effects of observed confounding, although the imbalance of the covariate remains a concern, particularly variables with extreme weights at the tails of the propensity score distribution (Fig. 2) [21]. Therefore, we further used CBPS to mitigate the imbalance among covariates and found that the strength of association between anaemia within the first week and one-year mortality between IPTW and CBPS appear to be similar, indicating that the covariates between the two groups should be largely comparable in the present study [20]. Taken together, we used four statistical analyses to demonstrate a consistent association between week-1 anaemia and one-year mortality in critically ill patients, and the slight difference in the strength of association should reflect the distinct statistical approaches and assumptions.

## Limitations

There are limitations in this study. First, this study is a single-centre study in central Taiwan, and more studies are warranted to validate our findings. Second, selection bias could be a concern due to the retrospective study design, but we have performed the propensity score-based matching and weighting analyses to guarantee the robustness of our results. Third, potential unmeasured confounders, such as alcohol intake and fluctuation of haemoglobin level, cannot be assessed; however, the adjustment for numerous variables and propensity score-based approach should at least partly mitigate this concern. Moreover, studies focusing on critically ill patients with moderate/severe liver diseases are warranted.

## Future directions

Our findings shed light on the long-term relevance of anaemia and provide clinical evidence for risk stratification of the long-term mortality by week-1 variables in critically ill patients. More studies are warranted to clarify underlying biological mechanisms and to explore the potential effect of correction of anaemia within and after the first week of ICU admission.

## Conclusions

In conclusion, anaemia is a substantial issue in critical illness, but the long-term effect on mortality of anaemia remains a research niche. In the present study, we linked the hospital-based critical care database at TCVGH with the Taiwanese nationwide death registry profile as well as propensity score-based methods to demonstrate that anaemia within the first week was associated with an increased risk of long-term mortality in critically ill patients.

## Electronic supplementary material

Below is the link to the electronic supplementary material.

Supplementary Material 1

## Data Availability

The data generated and analysed in this study are available from the corresponding author upon reasonable request.
