# Supplementary material for: Anaemia in the first week may be associated with long-term mortality among critically ill patients: propensity score-based analyses
Source: BMC Emerg Med. 2023 Mar 22;23:32. doi: 10.1186/s12873-023-00806-w (PMC10035173; doi:10.1186/s12873-023-00806-w)
Supplement: Supplementary file 1 — Supplementary Material 1 [file 12873_2023_806_MOESM1_ESM.pdf]

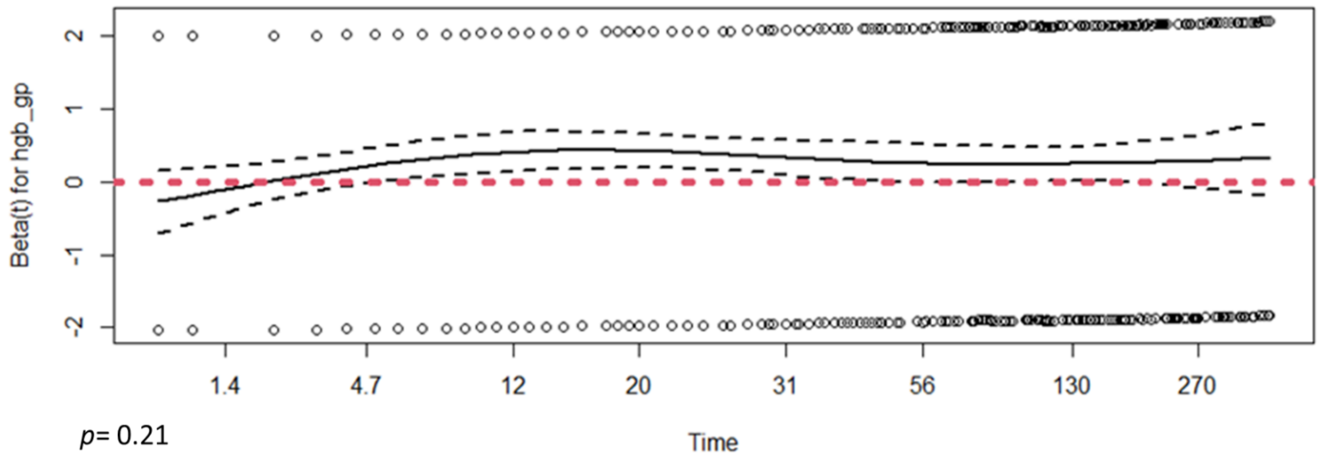

p= 0.21

Supplemental Figure 1. Schoenfeld residual plot to illustrate the correlation between the Schoenfeld residuals and survival time.

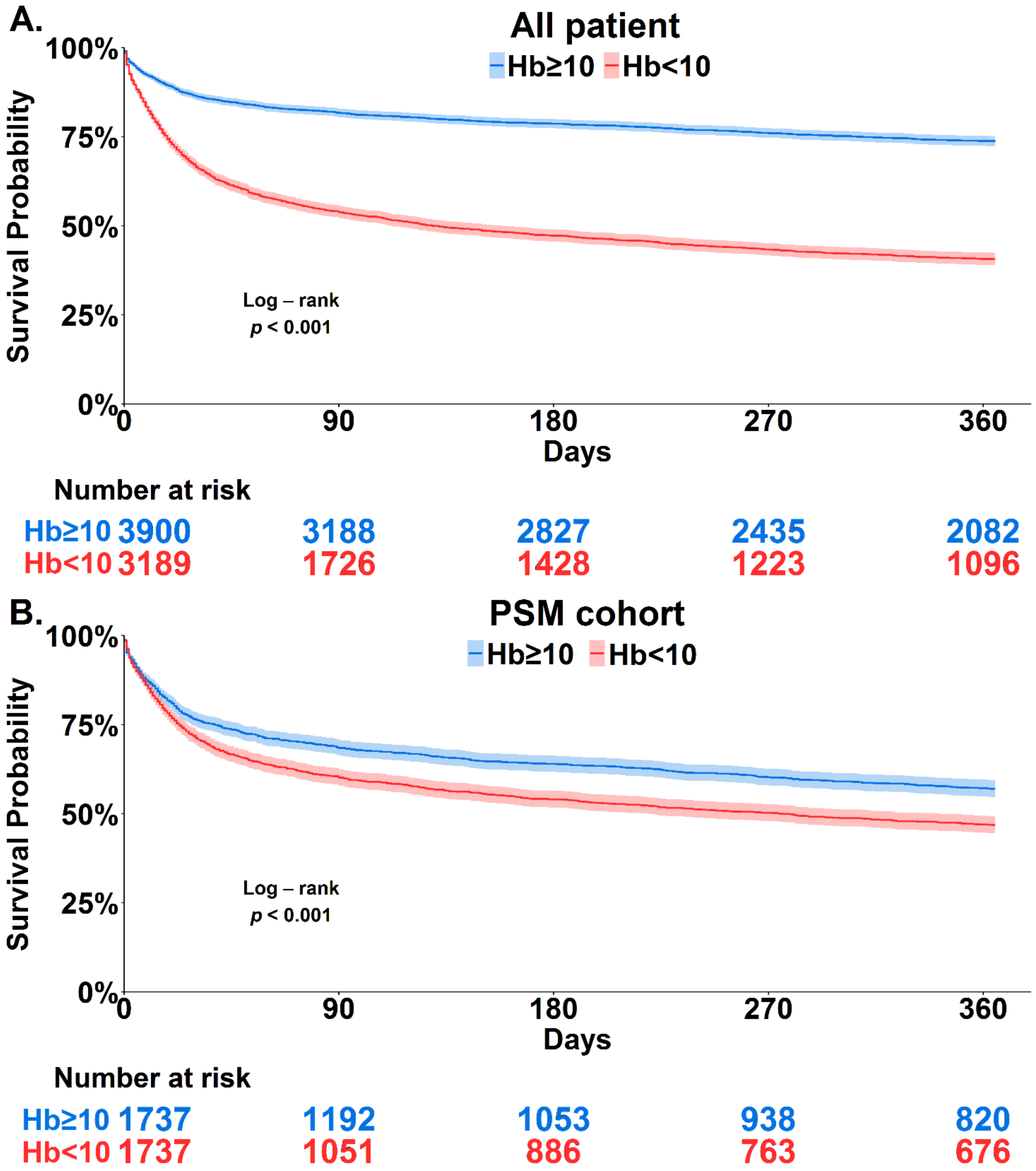

Supplemental Figure 2. Kaplan-Meier survival curves of critically ill patients whose week-1 hemoglobulin higher and lower than 10 g/dL. (A) primary cohort with all of enrolled subjects, (B) propensity score-matched cohort. Abbreviations: Hb, haemoglobin (g/dL); PSM, propensity score-matching.
